# Self-Compliance and High Performance Pt/HfO_x_/Ti RRAM Achieved through Annealing

**DOI:** 10.3390/nano10030457

**Published:** 2020-03-04

**Authors:** Lei Wu, Hongxia Liu, Jinfu Lin, Shulong Wang

**Affiliations:** Key Laboratory for Wide-Band Gap Semiconductor Materials and Devices of Education, School of Microelectronics, Xidian University, Xi’an 710071, China

**Keywords:** self-compliance, RRAM, annealing, HfOx, switching mechanism

## Abstract

A self-compliance resistive random access memory (RRAM) achieved through thermal annealing of a Pt/HfO_x_/Ti structure. The electrical characteristic measurements show that the forming voltage of the device annealing at 500 °C decreased, and the switching ratio and uniformity improved. Tests on the device’s cycling endurance and data retention characteristics found that the device had over 1000 erase/write endurance and over 10^5^ s of lifetime (85 °C). The switching mechanisms of the devices before and after annealing were also discussed.

## 1. Background

Resistance random access memory is considered one of the most promising candidates for the next generation of non-volatile memory due to its simple structure, highest storage capacity with the smallest cell size of 4F^2^ (F is the minimum feature size), fast switching speed, low power consumption, and compatibility with CMOS processes [1,2,3,4]. RRAMs based on various materials, including metal oxides [5,6], novel nanomaterials [7,8,9], and organics [10,11], have been proposed. Among them, RRAM based on hafnium oxide [12,13,14,15], which replaced SiO_2_ as the gate dielectric of choice below the 45 nm node [16,17], has been widely studied due to its high switching stability. There are still many challenges to overcome before RRAMs can be used as commercial memory, such as switching uniformity, overshoot current during forming or set process, leakage current in integrated arrays, and so on [4,18].

To prevent the overshoot current during forming or set process, additional current limits are usually imposed on the device [4,5], which adds to the complexity of the measuring circuit. In order to obtain devices with self-compliance characteristics, many schemes have been proposed, including the one diode and one resistor (1D1R) [19], and the one transistor and one resistor (1T1R) [20]. However, these increase the complexity of the device structure and the difficulty of fabrication. Self-compliance RRAM based on Ag/HfO_2_/Au structure was also proposed [21], but only 30 cycles of endurance were demonstrated. Therefore, research on devices with simple structures and self-compliance current characteristics is necessary. In this paper, a self-compliance RRAM device with high switching performance was proposed by annealing of a Pt/HfO_x_/Ti structure. It is a capacitive structure with only a single oxide medium as the switching layer and two metal layers as the electrodes, and the self-compliance characteristics can be obtained by just one step annealing at the end of the process, no additional limiting devices such as diodes or transistors are required, so the device preparation process can be simple. The simple device structure also allows for a high integration density.

## 2. Methods

The 20 nm Ti adhesion layer and the 100 nm Pt bottom electrodes (BE) deposited on Si substrates by electron beam vapor deposition. The BE patterns were obtained by lithography and lift-off process. Then, the HfO_x_ active layer deposited by the atomic layer deposition (ALD) method (Picosun R-150, Espoo, Finland). The temperature of the ALD chamber was set as 300 °C, and tetrakis (ethylmethylamino) hafnium (TEMAH) was used as the hafnium precursor, with H_2_O used as the oxidant. For an ALD growth cycle of HfO_x_, TEMAH was pulsed into the chamber by carrier gas for 0.3 s with an 8 s N_2_ purge, and then H_2_O was pulsed for 0.1 s, followed by an 8 s N_2_ purge. The HfO_x_ film thickness was measured by a spectral ellipsometer (J. A. Woollam Company M-2000D, Lincoln, NE, USA). Devices with 5 nm, 10 nm, 15 nm, and 20 nm thick HfO_x_ were prepared. The active layer pattern obtained by inductively coupled plasma (ICP) etching (Oxford Instruments Plasmalab System133, Oxford, UK). Finally, the 100 nm Ti top electrode (TE) and a 20 nm Pt cladding layer deposited by electron beam vapor deposition, followed by lithography and lift-off process. The effective areas of the devices depend on the upper and lower electrode wire widths, about 2 × 2 μm^2^, 6 × 6 μm^2^, 10 × 10 μm^2^, and 20 × 20 μm^2^ in this work. The as-deposited devices (Device A) were annealed in N_2_ (99.996%) for 10 min at 500 °C (Device B) and 600 °C (Device C) in a rapid thermal processing (RTP) system (UniTemp GmbH RTP 100, Pfaffenhofen, Germany). Electrical characteristics measured by the semiconductor device analyzer (Agilent B1500A, Palo Alto, CA, USA) with a source/measurement unit and a waveform generator/fast measurement unit. The Ti TE was biased, while the Pt BE was grounded during the whole measurement.

## 3. Results and Discussion

**Figure 1:**
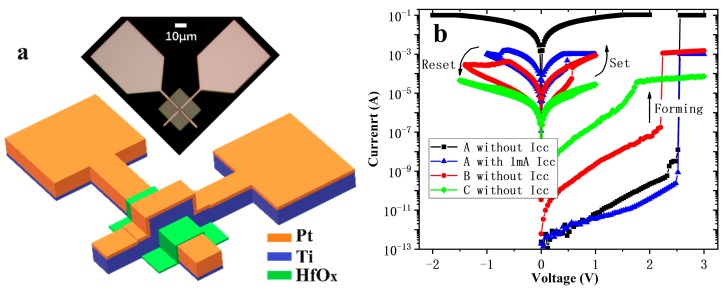
The structure and I-V characteristics of the 2 × 2 μm^2^ devices with 10 nm thick HfO_x_. (**a**) the structure diagram and optical micrograph of the devices; (**b**) forming and typical set/reset characteristics of Device A, B, and C.

Figure 1a shows the structure diagram and optical micrograph of the device. The fresh devices with a thickness of HfO_x_ film more than 5 nm need a soft breakdown (named forming) before normal cycles, by applying a large positive voltage to make the device from high resistance state (HRS) to low resistance state (LRS). In normal cycles, a negative voltage was applied to the device and reset it from LRS to HRS, and a positive voltage set it from HRS to LRS, as shown in Figure 1b. The as-deposited devices do not have self-compliance characteristics. The forming current of the devices will reach the maximum allowable current of the instrument (100 mA) if an additional compliance current (Icc) is not applied, which will be damaged and unable for the devices to reset from LRS to HRS. After thermal annealed at 500 °C for 10 min, the initial resistance and the forming voltage of the devices reduced, and showed the characteristic of self-compliance. As for the devices annealed at 600 °C, the initial resistance and the forming voltage also reduced, and they also show self-compliance characteristic, but the resistance ratio of the devices was too small to act as resistance switching. The possible reason for the degradation of Device C may be the transformation of HfOx from amorphous to crystalline state after annealing at 600 °C for 10 min [16,22,23].

**Figure 2:**
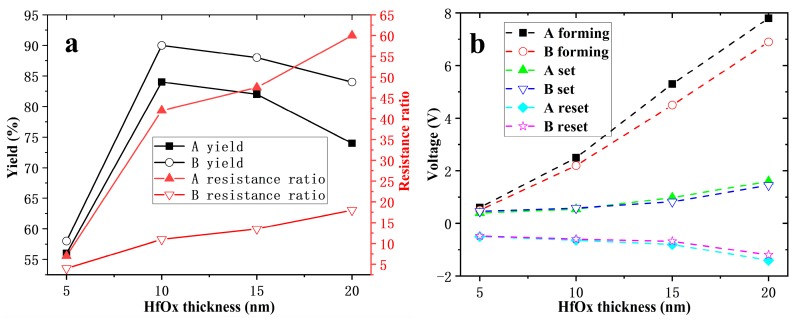
HfO_x_ thickness dependence of yield, resistance ratio (**a**) and operating voltages (**b**).

The effect of different HfO_x_ thickness on yield is shown in Figure 2a as black lines. 50 devices were tested for each thickness. The yield of devices annealed at 500 °C is higher than that of unannealed, and indicates that the annealing step helps to improve the yield of devices. The yield increases first, and then decreases with increasing HfO_x_ thickness, and the devices annealed at 500 °C have the highest yield, about 92%. HfO_x_ thickness dependence of resistance ratio and operating voltages were studied. Each parameter was averaged for 20 qualified devices. The red line in Figure 2a shows that the resistance ratio increases as the HfO_x_ thickness increases. The resistance ratio of 500 °C annealed devices is higher than that of unannealed devices, and this improvement is particularly significant for devices over 10 nm Hf. As shown in Figure 2b, the voltage amplitudes required for forming, set, and reset all increase with the thickness of HfO_x_. For the devices with 5 nm HfO_x_, the forming voltage is approximately the set voltage, which means it is forming-free. The forming voltage of devices annealed at 500 °C is lower than that of unannealed devices, but this change is not obvious in set and reset voltages.

**Figure 3:**
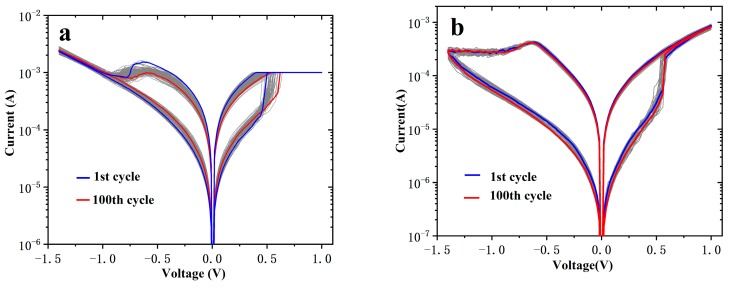

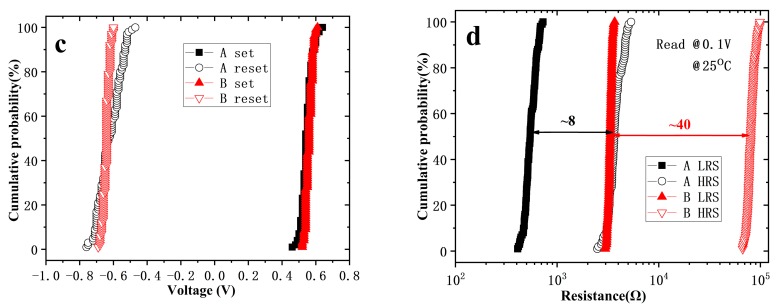
I-V characteristics of the first 100 DC scans of 2 × 2 μm^2^ and 10 nm thick HfO_x_ devices without annealing (**a**) and annealed at 500 °C (**b**), and their accumulative probability of set/reset voltages (**c**) and HRS/LRS (**d**).

Figure 3 shows the I-V curves of the first 100 cycles of Device A and B by DC sweep. After 100 cycles, the reset voltage of Device A decreased while the set voltage increased, and the switching ratio degraded. As for Device B, the set/reset voltage barely changed, while the resistance ratio improved a little. The set/reset voltage and HRS/LRS distribution of Device A and B are exhibited in Figure 3c,d, respectively. After annealing at 500 °C for 10 min, the uniformity of the set/reset voltage and HRS/LRS are greatly improved, and the switching ratio has increased from about 8 to about 40.

**Figure 4:**
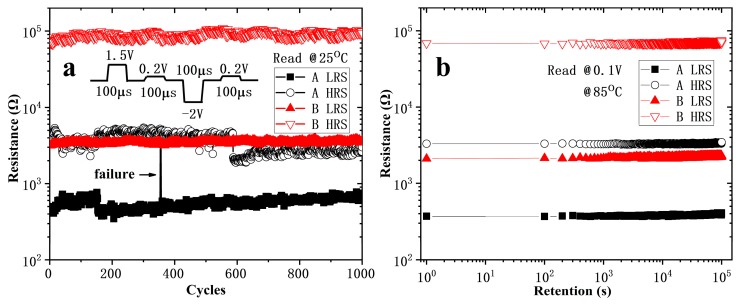
Cycling endurance (**a**) and data retention (**b**) at 85 °C for Device A and B. The area of the devices is 2 × 2 μm^2^ and the thickness of HfO_x_ is 10 nm.

The erase/write endurance of the devices tested with a waveform generator/fast measurement unit of B1500A in a voltage pulses mode. As shown in Figure 4a, the voltage amplitudes of set, reset, and read pulse are 1.5 V, −2 V, and 0.2 V, respectively. All the pulse widths and the intervals between pulses are 100 µs. The resistance values of Device A fluctuated greatly, and there were failure operations occurred. The resistance values in both states of Device B were stable and no significant degradation of switching ratio observed after 1000 cycles. Figure 4b shows the date retention at 85 °C, indicating a lifetime over 10^5^ s is expected for both Device A and B.

**Figure 5:**
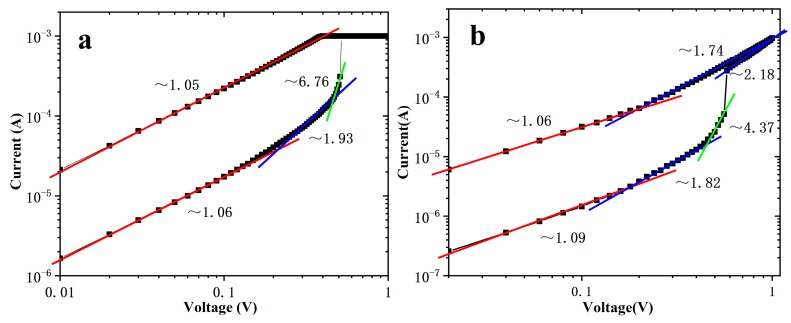
I-V characteristics of (**a**) Device A and (**b**) Device B in a double-logarithmic plot. The area of the devices is 2 × 2 μm^2^ and the thickness of HfO_x_ is 10 nm.

To clarify the switching mechanisms of the devices, the I-V curves of set process were replotted in double logarithmic scale in Figure 5. For Device A, according to space charge limited current (SCLC) mechanism [24,25,26], the I-V characteristics in the low positive bias region exhibit ohmic conduction (I∝V) because much of the space charges injected into HfO_x_ are trapped by the empty trapping levels in HfO_x_ and are unable to contribute to current. As the electric field increases, the number of injected excess carriers increases, and the trapping energy level is gradual filled, so the injected carriers are dominating over the thermal-generated carriers. It is called Child’s law region (I∝V^2^). The steep current increase region (I∝V^6.76^) appears when the conducting filaments form between the electrodes. At LRS, it follows ohmic conduction mechanism. The conductive mechanism of Device B is similar to that of Device A at HRS. The difference is that, in the high bias region, Child’s law region is observed again after the steep current increase region. I-V characteristics in LRS exhibit trap-unfilled SCLC conductivity: As the sweep voltage decreases, there is a transition region (I∝V^1.74^) from Child’s law to ohmic conduction in high bias region, and then ohmic conduction (I∝V^1.06^) in low bias region.

**Figure 6:**
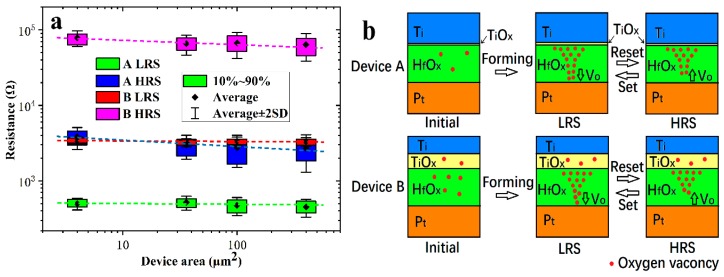
(**a**) Device area dependence of resistance of 10 nm HfO_x_ devices and (**b**) schematic description of the resistive switching mechanism.

To better identify the switching mechanism, a junction-size dependence of device resistance was shown in Figure 6a. The data for each state was counted from 20 devices. The resistance of the LRS is independent of the device cell area, indicating the LRS is dominated by localized conducting path [11,19]. For HRS, the resistance was shown as slightly inversely proportional to the cell area, which is attributed to the homogeneous current flowing through the broken part of the conducting filament, and the conducting filament is still partially present. Figure 6a also showed significant device to device variability of resistance for devices without annealing, but the variability decreased for devices annealed at 500 °C.

Based on the above analysis, the possible switching mechanisms of the devices are summarized in Figure 6b. For Device A, as the positive voltage applied to the titanium electrode increases, more and more oxygen ions are generated in the HfO_x_ and move toward the titanium electrode [27], producing titanium oxide. At the same time, the oxygen vacancies accumulate toward the interface of HfO_x_/Pt and form conductive filaments gradually [28]. The device turns to LRS when the oxygen vacancies conducting filaments connect the TE and BE. When the titanium electrode is applied with a negative voltage, the oxygen ions combine with the oxygen vacancies at the interface of HfO_x_/Pt [29,30], which leads to the partial rupture of the conductive filament, and the device resets to HRS. When a positive bias is applied to the TE, the conducting filaments reconnect, and the device set to LRS again.

The initial state of Device B is quite different from that of Device A. As Ti tends to react easily with oxygen atoms in transition metal oxides for its low standard free energy of formation of oxides [31], after annealing at 500 °C for 10 min, the top electrode Ti capture the oxygen atoms from HfO_x_ and form a TiO_x_ layer at the interface [32]. Similar situations have been reported in [33] using AlCu as the electrode. Due to the similar conductive behavior of Device A and B at HRS in Figure 4, we assume that the switching layer of Device B is also located at the HfO_x_ layer. However, compared to that of Device A, the TiO_x_ layer of Device B is thick enough to limit the overshoot current as a series resistor. The trap-unfilled SCLC conductivity mechanism of Device B at LRS is also explained, which is caused by the unbroken TiO_x_. Due to the voltage division of the series resistor, the uniformity and stability of the device are improved. At the beginning of forming/set, the resistance value of the switching layer is much higher than the series resistor, according to Ohm’s law, almost all the voltage is applied to the switching layer. When the voltage increases to the forming/set threshold voltage, the device changes to LRS, and the resistance of the switching layer approaches that of the series resistor. The voltage applied to the switching layer is reduced by the partial voltage action of the series resistor, avoiding the voltage on the switching layer being too large to shoot it down. It also prevents the switching layer from forming/set to a too low resistance state due to the continuously increasing voltage, which improve the uniformity of the resistance of LRS. A too low resistance state also means the conductive path in the device is too strong to reset back to HRS, which increases the possibility of operation failure (as shown in Figure 4a). In addition, since the Ti captures the oxygen ions from HfO_x_, the oxygen vacancy concentration in HfO_x_ increases. High-density oxygen vacancies make for the formation of CF [3,7] which can be observed from the decreased forming voltage. High-density oxygen vacancies also increase the uniformity of charge carriers’ distribution, and undoubtedly strengthen the CF and enhance its stability. In this way the behavior of CF is improved, and the stability of resistance in both the LRS and the HRS is improved, as shown in Figure 4a.

## 4. Conclusion

A self-compliance Pt/HfOx/Ti RRAM obtained by annealing at 500 °C for 10 min in N_2_. The forming voltage and operating current of the annealed devices reduced, meanwhile, the device to device variability, and the stability and the resistance ratio improved. The erase/write testing by pulse mode and data retention testing indicate that the endurance of more than 1000 cycling and a lifetime of over 10^5^ s at 85 °C is expected. Although the accurate switching mechanisms still need to be further studied, through the analysis of the device area dependence of resistance and the conductive mechanism, we made suggestions that self-compliance characteristics and performance improvement of the annealed device may be due to the gathering O of the top electrode Ti from HfO_x_, and forming a thick layer of TiO_x_ at the Ti/HfO_x_ interface. The annealing step to obtain self-compliance characteristics and improve device performance avoid the additional current limiting devices like diodes and transistors, so it simplifies the fabrication process, and makes the device realize the smallest cell size of 4F^2^.
